# The ecology of anaerobic degraders of BTEX hydrocarbons in aquifers

**DOI:** 10.1093/femsec/fiw220

**Published:** 2016-12-10

**Authors:** Tillmann Lueders

**Affiliations:** Institute of Groundwater Ecology, Helmholtz Zentrum München–German Research Center for Environmental Health, 85764 Neuherberg, Germany

**Keywords:** toluene, benzene, groundwater, fumarate-adding enzymes, stable isotope probing, degrader diversity

## Abstract

The degradation of benzene, toluene, ethylbenzene and xylene (BTEX) contaminants in groundwater relies largely on anaerobic processes. While the physiology and biochemistry of selected relevant microbes have been intensively studied, research has now started to take the generated knowledge back to the field, in order to trace the populations truly responsible for the anaerobic degradation of BTEX hydrocarbons *in situ* and to unravel their ecology in contaminated aquifers. Here, recent advances in our knowledge of the identity, diversity and ecology of microbes involved in these important ecosystem services are discussed. At several sites, distinct lineages within the *Desulfobulbaceae*, the *Rhodocyclaceae* and the Gram-positive *Peptococcaceae* have been shown to dominate the degradation of different BTEX hydrocarbons. Especially for the functional guild of anaerobic toluene degraders, specific molecular detection systems have been developed, allowing researchers to trace their diversity and distribution in contaminated aquifers. Their populations appear enriched in hot spots of biodegradation *in situ*. ^13^C-labelling experiments have revealed unexpected pathways of carbon sharing and obligate syntrophic interactions to be relevant in degradation. Together with feedback mechanisms between abiotic and biotic habitat components, this promotes an enhanced ecological perspective of the anaerobic degradation of BTEX hydrocarbons, as well as its incorporation into updated concepts for site monitoring and bioremediation.

## Teaser

This review summarises recent advances into the identity, diversity and ecology of anaerobic degraders of monoaromatic hydrocarbons in contaminated aquifers.

## INTRODUCTION: BTEX CONTAMINATION OF GROUNDWATER SYSTEMS

Contamination with aromatic hydrocarbons, specifically benzene, toluene, ethylbenzene and xylenes (BTEX), is of major concern to groundwater quality and aquifer ecosystem health. BTEX compounds are prominently placed amongst the US Agency for Toxic Substances and Disease Registry's list of priority pollutants, based on their frequency, toxicity and potential for human exposure (ATSDR 2007). BTEX contamination of the subsurface is often a legacy of petroleum or gasoline processing, transport and storage, of coal gasification, chemical hydrogenation or other industrial activities and accidents (Wilkes and Schwarzbauer 2010).

**Figure 1.**
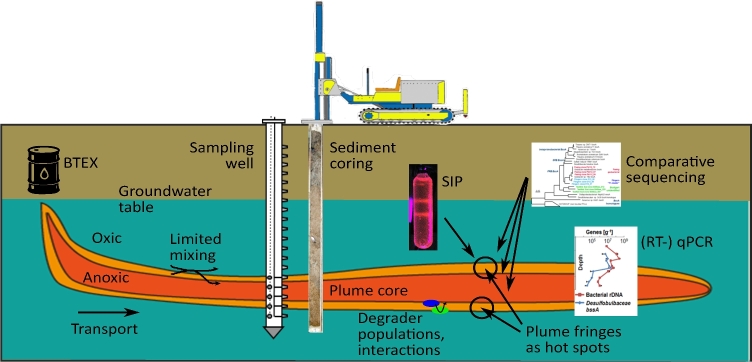
Conceptual view of longitudinal plume zonation in a BTEX-contaminated aquifer. The plume core is depleted in electron acceptors, while degraders and degradation activities reside especially at the plume fringes. Sampling possibilities for groundwater and sediments are indicated, same as major factors influencing degrader ecology and approaches to investigate them, as discussed in the text.

Since BTEX compounds are relatively soluble and mobile in water and either carcinogenic or neurotoxic to humans, it is relevant to understand and predict their fate in groundwater systems. BTEX contaminations typically spread as plumes, mobilised by advective transport and dispersive mixing downstream of the source (Fig. 1). However, such contaminants are known to be naturally attenuated in aquifers via processes including dilution, dispersion, sorption, abiotic transformation and most important, biodegradation. The latter is considered as the only sustainable component of natural attenuation (Kleinsteuber, Schleinitz and Vogt 2012; Meckenstock *et al.*^2015^).

In contrast to many contaminated soils and surface waters, the saturated subsurface is limited in oxygen replenishment. Hence, increased organic carbon input by contamination results in a rapid depletion of available oxygen. The maximal oxygen concentration of ∼8 mg l^−1^ (0.5 mM) in pristine groundwater suffices to oxidise a toluene load of ∼5 mg l^−1^ (∼0.05 mM), much less than what is often found at contaminated sites. Thus, contaminated aquifers are typically reduced and biodegradation depends on anaerobic processes. The classical perspective of such systems involves a reverse longitudinal redox zonation of contaminant plumes, ranging from methanogenic/fermenting conditions in zones of highest contamination to the reduction of energetically more favourable electron acceptors such as sulphate, ferric iron or nitrate along the flow path (Christensen *et al.*^2000^). While this perspective has been developed towards a greater importance of redox gradients and biodegradation at the plume fringes (Meckenstock *et al.*^2015^), anaerobic degradation and degraders of BTEX compounds for all respective respiratory processes have been reported (Table 1).

**Table 1.** Thermodynamics of toluene oxidation under aerobic and anaerobic respiration and in syntrophy^a^.

Electron acceptor		Δ*G*^0΄^
(oxidised/reduced)	Stoichiometry	[kJ (mol C_7_H_8_)^−1^]
O_2_ / H_2_O	C_7_H_8_ + 9 O_2_ + 3 H_2_O → 7 HCO_3_^−^ + 7 H^+^	−3790 kJ
NO_3_^−^/N_2_	5 C_7_H_8_ + 36 NO_3_^−^ + H^+^ → 35 HCO_3_^−^ + 18 N_2_ + 3 H_2_O	−3555 kJ
Fe(OH)_3_/FeCO_3_	C_7_H_8_ + 36 Fe(OH)_3_ + 29 HCO_3_^−^ + 29 H^+^ → 36 FeCO_3_ + 87 H_2_O	−1497 kJ
SO_4_^2^^−^/HS^−^ (complete)^b^	2 C_7_H_8_ + 9 SO_4_^2−^+ 6 H_2_O → 14 HCO_3_^−^ + 9 HS^−^ + 5 H^+^	−203 kJ
		−45 kJ (mol SO_4_^2−^)^−1^
SO_4_^2^^−^ HS^−^ (incomplete)^b^	2 C_7_H_8_ + 3 SO_4_^2−^+6 H_2_O → 6 CH_3_COO^−^ + 2 HCO_3_^−^ + 3 HS^−^ + 5 H^+^	−61 kJ
		−41 kJ (mol SO_4_^2−^)^−1^
CO_2_/CH_4_ (sum)	2 C_7_H_8_ + 15 H_2_O → 9 CH_4_ + 5 HCO_3_^−^ + 5 H^+^	−130 kJ
Fermenter	2 C_7_H_8_ + 18 H_2_O → 6 CH_3_COO^−^ + 2 HCO_3_^−^ + 8 H^+^ + 12 H_2_	+166 kJ
Hydrogenotroph	12 H_2_ + 3 HCO_3_^−^ + 3 H^+^ → 3 CH_4_ + 9 H_2_O	−203 kJ
Acetotroph	6 CH_3_COO^−^ + 6 H_2_O → 6 CH_4_ + 6 HCO_3_^−^	−93 kJ

a Toluene is highlighted as representative BTEX compound here. The table has been adapted from Weelink *et al.* (2010) and Widdel *et al.* (2010).

b Complete or incomplete oxidation of toluene.

## ANAEROBES CAPABLE OF OXIDISING BTEX COMPOUNDS

Early research on the degradation of BTEX hydrocarbons was mostly directed towards aerobic degraders and degradation pathways (Zylstra and Gibson 1991; Pérez-Pantoja, González and Pieper 2010). Recent years have seen a substantial increase in the number of isolated anaerobic degraders and enrichment cultures, as well as in physiological and biochemical insights into involved catabolic pathways. As these advances have been intensively reviewed (Weelink, van Eekert and Stams 2010; Widdel, Knittel and Galushko 2010; Fuchs, Boll and Heider 2011; Rabus *et al.*^2016^), it is not the intention of this article to expand on this in detail. Instead, it briefly synthesises the fundamentals to then focus on how researchers have taken this knowledge back to the field, in order to identify the microbes truly responsible for anaerobic degradation *in situ* and to unravel their ecology in contaminated aquifers. Specific attention is given to the study of anaerobic toluene-degrading populations, for which the most significant ecological advances have been achieved.

### Cultures and enrichments

Most anaerobic degraders of monoaromatic compounds isolated to date are members of the *Betaproteobacteria*, *Deltaproteobacteria* and *Clostridia*. Under denitrification, a number of *Azoarcus*, *Thauera* and ‘*Aromatoleum’* spp. and other closely related *Rhodocyclaceae* are known to degrade toluene and/or ethylbenzene or xylenes (see lists in Weelink, van Eekert and Stams 2010; Widdel, Knittel and Galushko 2010). Within the *Alphaproteobacteria*, members of the genus *Magnetospirillum* (*Rhodospirillaceae*) can also oxidise toluene while respiring nitrate (Shinoda *et al.*^2005^). Denitrifying *Azoarcus* and *Dechloromonas* strains were actually the first isolates suggested to degrade benzene under anaerobic conditions (Coates *et al.*2001b; Kasai *et al.*^2006^). More recent evidence from denitrifying benzene-degrading enrichments also indicates an involvement of members of the *Peptococcaceae* (*Clostridia*) in this process (van der Zaan *et al.*^2012^; Luo *et al.*^2014^).

Under iron-reducing conditions, several *Geobacter* spp. (*Geobacteraceae*) are known to degrade toluene or xylenes (Coates *et al.*2001a; Kunapuli *et al.*^2010^; Prakash *et al.*^2010^), as are the betaproteobacterial *Georgfuchsia toluolica* (*Rhodocyclaceae*, Weelink *et al.*^2009^) and the clostridial *Desulfitobacterium aromaticivorans* (*Peptococcaceae;* Kunapuli *et al.*^2010^). *Geobacter metallireducens* has also been proven to degrade benzene under iron reduction (Zhang *et al.*^2012^). All of these iron-reducing degraders of monoaromatic hydrocarbons have been isolated from terrestrial samples, which discriminates them from denitrifying degraders where only a few (Kasai *et al.*^2006^) are of non-marine origin. Anaerobic benzene degradation has also been shown for the hyperthermophilic, iron-reducing Archaeon *Ferroglobus placidus* (Holmes *et al.*^2011^), yet the relevance of such thermophilic degraders may be limited to very specific habitats.

Many sulphate-reducing degraders of BTEX compounds are related to *Desulfosarcina*, *Desulfobacula, Desulfotignum* spp. or to other *Desulfobacteraceae* and have been isolated from marine habitats (see tables in Weelink, van Eekert and Stams 2010; Widdel, Knittel and Galushko 2010). In contrast, the low number of mostly toluene-degrading sulphate reducers of terrestrial origin is phylogenetically more widespread. Here, monoaromatic hydrocarbon-degrading strains related to the deltaproteobacterial *Desulforhabdus* (*Syntrophobacteraceae*), *Desulfocapsa* (*Desulfobulbaceae*), and the clostridial *Desulfotomaculum* and *Desulfosporosinus* spp. (*Peptococcaceae*) have been isolated (Beller *et al.*^1996^; Meckenstock 1999; Liu *et al.*^2004^; Morasch *et al.*^2004^). The latter were the first non-proteobacterial anaerobic toluene degraders discovered. More recently, further sulfidogenic benzene- or TEX-degrading enrichment cultures dominated by microbes within the *Peptococcaceae* have been documented in samples from shallow (Kleinsteuber *et al.*^2008^; Abu Laban *et al.*^2009^) or deep contaminated aquifers (Berlendis *et al.*^2010^).

In the absence of all other electron acceptors, methanogenic oxidation of BTEX hydrocarbons occurs via syntrophy between fermenting degraders and methanogenic *Archaea* (Vogt, Kleinsteuber and Richnow 2011). Here, although defined co-cultures are still lacking, several highly enriched degrader cultures have been phylogenetically dissected. These cultures consistently harboured members of *Peptococcaceae*, *Deltaproteobacteria* and different hydrogenotrophic (*Methanospirillum*, *Methanobacterium* spp.) or acetotrophic (*Methanosaeta* spp.) methanogens, irrespective of whether they degraded toluene or benzene (Ficker *et al.*^1999^; Ulrich and Edwards 2003; Fowler *et al.*^2012^). Detailed functional insights into these enrichment cultures, e.g. as obtained by the use of stable isotope probing (SIP), will be discussed in later sections.

### Degradation pathways

**Figure 2.**
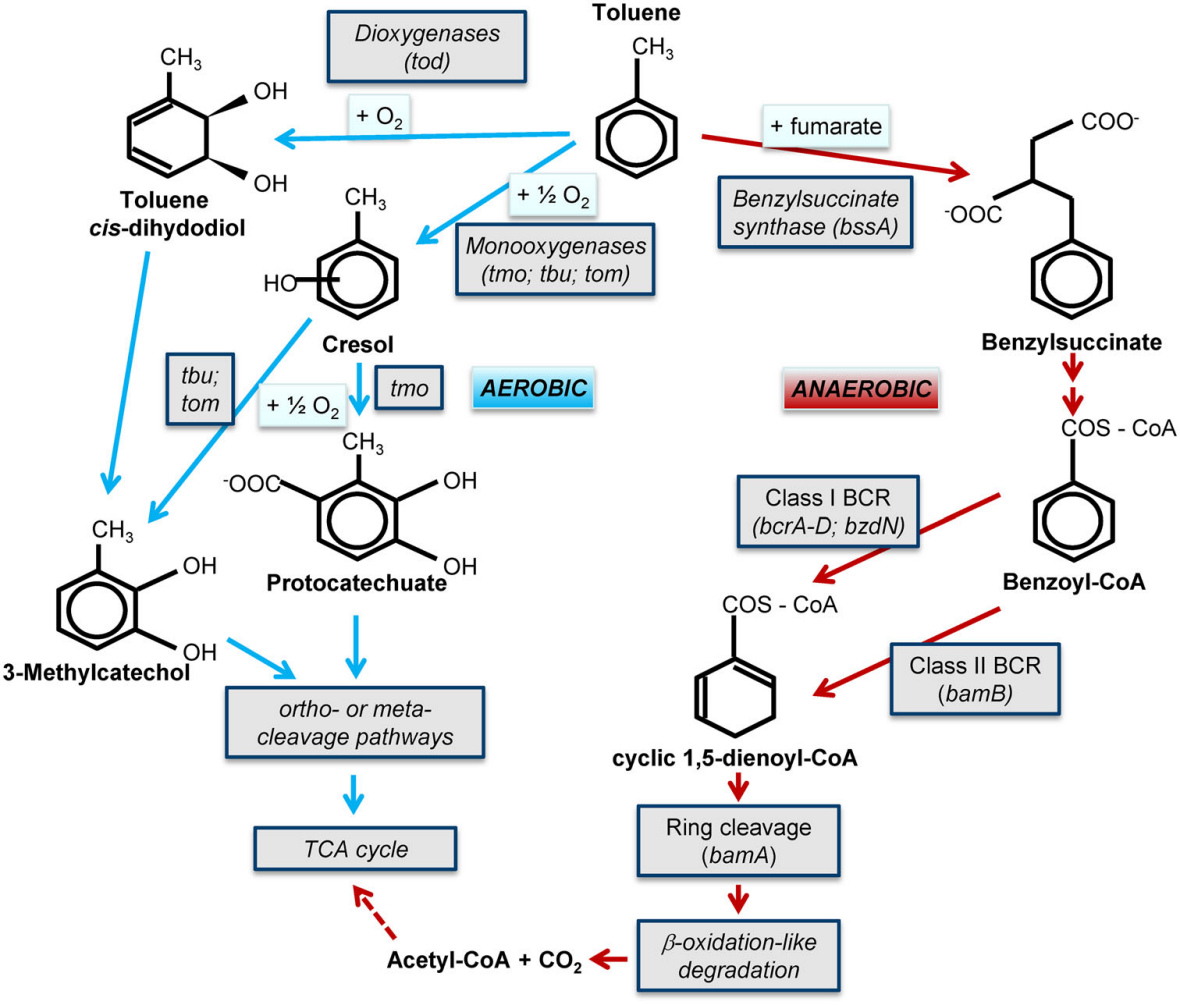
Initial activating reactions, peripheral and central biochemical pathways involved in the aerobic and anaerobic degradation of toluene as representative BTEX compound. Shown aerobic hydroxylation reactions represent the more conserved aerobic activation mechanisms. Important catabolic enzymes (*genes*) as mentioned in the text are given. Scheme synthesised from Parales *et al.* (2008) and von Netzer *et al.* (2016).

No matter which oxidant is respired, all degradation pathways of aromatic compounds proceed via conserved peripheral and central catabolic routes (Fuchs, Boll and Heider 2011). That is, the aerobic degradation of toluene proceeds via the initial addition of hydroxyl groups by distinct mono- or dioxygenases (Pérez-Pantoja, González and Pieper 2010), relying on molecular oxygen as a co-substrate to destabilise the aromatic ring (Fig. 2). Under anoxic conditions, the initial enzymatic attack of toluene is catalysed by the enzyme benzylsuccinate synthase (Bss), adding the methyl group of toluene to fumarate (so-called fumarate addition) forming benzylsuccinate (Fuchs, Boll and Heider 2011; Rabus *et al.*^2016^). This enzymatic step involves a glycyl-radical mechanism, explaining how chemically stable aromatic compounds such as toluene can be activated in the absence of molecular oxygen. Benzylsuccinate is then further activated as a CoA-thioester, which is then transformed, via several intermediates, to benzoyl-CoA. The latter is an important central intermediate in the anaerobic catabolism of aromatic compounds, funnelling metabolites from diverse peripheral degradation pathways into aromatic ring reduction, ring cleavage and finally a modified β-oxidation to acetyl-CoA (Fuchs, Boll and Heider 2011; Rabus *et al.*^2016^). The first step of ring reduction can either be conducted by an ATP-dependent class I benzoyl-CoA reductase (BcrA-D) or an ATP-independent class II benzoyl-CoA reductase (BamB-I; Fuchs, Boll and Heider 2011). The former is typically found within facultative anaerobes, while the latter is hosted mostly by strict anaerobes and fermenters (Boll *et al.*^2014^). Subsequent ring cleavage by 6-oxocyclohex-1-ene-1-carbonyl-CoA hydrolase (*BamA*) is followed by β-oxidation-like reactions to acetyl-CoA for assimilation or complete oxidation to CO_2_ (Fig. 2). The degradation of xylenes and ethylbenzene also often involves fumarate addition as initial activation, though an alternative oxygen-independent hydroxylation has been reported for several denitrifying ethylbenzene degraders (Rabus *et al.*^2016^).

The initial enzymatic attack of benzene by anaerobic degraders is still much less understood. It may involve a carboxylation reaction to benzoate (Vogt, Kleinsteuber and Richnow 2011; Meckenstock *et al.*^2016^), which is then directly funnelled into central benzoyl-CoA catabolism. A distinct route for benzene catabolism is suggested for the facultatively anaerobic, denitrifying *Dechloromonas aromatica* (Coates *et al.* 2001). Instead of genes typically involved in anaerobic aromatics degradation, the genome of this *Betaproteobacterium* surprisingly contains several oxygenase genes usually found in aerobic aromatics degraders (Salinero *et al.*^2009^). This has given rise to the hypothesis of an oxygen-dependent, oxygenase-mediated attack of benzene under nitrate-reducing conditions (Weelink, van Eekert and Stams 2010). The activation mechanism, driven by self-sustained oxygenesis, can be considered analogous to methane oxidation as proposed for the NO-dismutating *Candidatus* Methylomirabilis oxyfera (Ettwig *et al.*^2010^), or to the oxygenase-dependent activation of benzene coupled to chlorite dismutation suggested for *D. aromatica* (Coates *et al.*2001b) and *Alicycliphilus denitrificans* (Weelink *et al.*^2008^). However, nitrate is a much more ubiquitous electron acceptor in groundwater than chlorite. In summary, anaerobic degraders of BTEX hydrocarbons have evolved a host of catabolic pathways allowing them to capitalise on hydrocarbons in the environment. However, only few of the involved genes and mechanisms have actually been targeted to trace respective populations and activities in the field.

## ANAEROBIC DEGRADERS OF BTEX HYDROCARBONS *IN SITU*

Over approximately the last decade, researchers have made intensive use of the so-called molecular ecology toolbox to trace anaerobic degraders of monoaromatic hydrocarbons in contaminated aquifers. An unambiguous identification of key players relevant at a distinct site allows elucidating their ecology and potential bottlenecks of biodegradation *in situ*. There are two major strategies: one involves the detection and phylogenetic affiliation of catabolic biomarkers (genes, transcripts, potentially proteins) in the field, including their quantification. The strength of this approach is that field samples are directly analysed, without prior laboratory handling. A drawback is that prior sequence knowledge of catabolic markers is essential for their detection. The second strategy is based on isotopic labelling of biomarkers (cellular lipids, nucleic acids, proteins, cells) via labelled contaminants, and the subsequent identification of labelled degraders. While this approach, due to the scarcity of biomass in aquifer samples, often requires laboratory incubation (and degrader enrichment to a certain extent), prior knowledge on degrader identity and involved catabolic pathways is not essential. Ideally, both directed (marker-based) and undirected (labelling-assisted) approaches should be combined to functionally and phylogenetically dissect degrader populations at a given site.

### Catabolic markers

Anaerobic degraders of BTEX aromatics are a catabolically defined but phylogenetically diverse functional guild, which cannot be comprehensively targeted via ribosomal markers. Thus, some of the conserved key enzymes in BTEX catabolism and their respective genes have been established as catabolic markers for degrader populations *in situ*. Most prominently, the benzylsuccinate synthase alpha-subunit (*bssA*) and related fumarate-adding enzyme (FAE) genes are applied for the detection of anaerobic alkylbenzene degraders (von Netzer *et al.*^2016^). Initially introduced as a qPCR assay for denitrifying toluene degraders within the *Betaproteobacteria* (Beller *et al.*^2002^), increasing pure culture sequence availability has allowed for the development and continuous improvement of primers and detection assays for degraders active in iron-reducing, sulphate-reducing and methanogenic environments. A comprehensive overview of the assays available is beyond the scope of this review and published elsewhere (von Netzer *et al.*^2016^).

**Figure 3.**
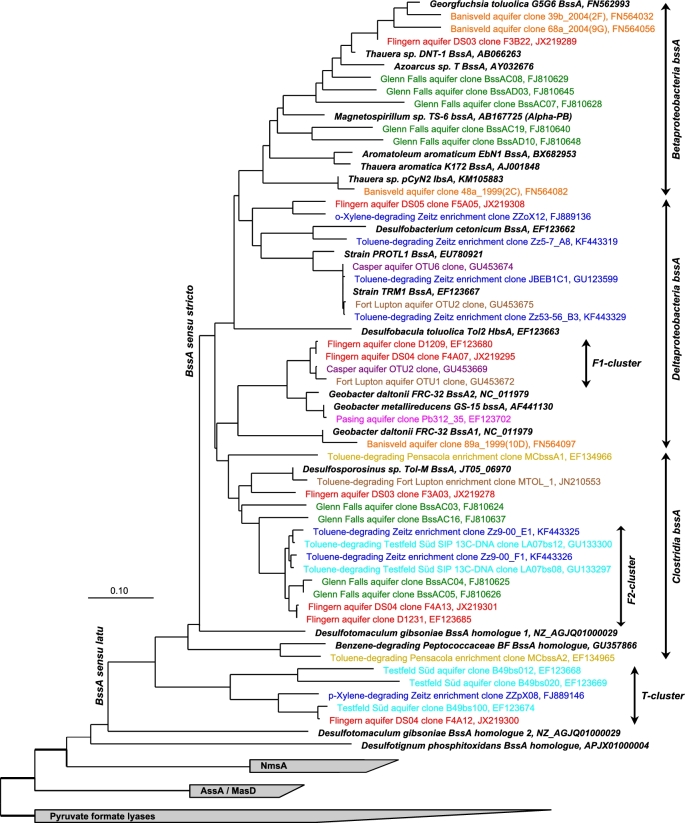
Overview of the phylogeny of *bssA* genes and close homologues for FAEs. Environmental sequences retrieved from different hydrocarbon-contaminated aquifers and enrichment cultures are given in different colours. Major gene lineages as mentioned in the text are named. Outgroup: related pyruvate formate lyase genes. Tree adapted from and reconstructed as in von Netzer *et al.* (2013, 2016).

In the field, targeted catabolic gene approaches for anaerobic toluene degradation were first applied for a number of tar oil-contaminated aquifers in Germany (Winderl, Schaefer and Lueders 2007). The authors revealed several unidentified and potentially site-specific degrader lineages, especially at sites with sulphate reduction (Fig. 3). The novel ‘F1-cluster’ *bssA* genes, more closely related to geobacterial *bssA* than that of other sulphate reducers, dominated a former gasworks site in Flingern, and was quantitatively enriched at the sulfidogenic lower fringe of the toluene plume (Winderl *et al.*^2008^). This suggested a potential of catabolic marker quantification to identify hot spots of biodegradation *in situ*. However, more deeply branching *bssA* homologues of unclear affiliation were also recovered, such as the ‘T-cluster’ detected at the Testfeld Süd aquifer near Stuttgart (Winderl, Schaefer and Lueders 2007). The diversity of *bssA* genes and homologues was next analysed at the hydrocarbon-contaminated Fort Lupton (Colorado) and Casper (Wyoming) aquifers (Callaghan *et al.*^2010^). At both sites, specific populations of mostly desulfobulbal *bssA* sequence types were identified (Fig. 3). A considerable diversity of both betaproteobacterial and more deeply branching *bssA* genes was found at the coal tar-contaminated South Glens Falls aquifer, New York (Yagi *et al.*^2010^). In contrast, rather limited diversities of mostly betaproteobacterial *bssA* sequence types were found enriched within a landfill-leachate plume at the Banisveld aquifer, the Netherlands, most of them related to *Georgfuchsia toluolica* (Staats, Braster and Röling 2011). In fact, this iron reducer had originally been isolated from the same site (Weelink *et al.*^2009^), thus strengthening the link between studies performed in lab and field.

A qPCR assay designed to detect *bssA* genes of sulphate-reducing and syntrophic alkylbenzene degraders was used to monitor degrader abundance and dynamics in two artificially contaminated well galleries at the Vandenberg Air Force Base, California, one of them additionally amended with ethanol (Beller *et al.*^2008^). Higher comparative *bssA* abundance was observed under ethanol amendment, indicating that this could stimulate degrader abundance during bioremediation. Comparative qPCR assays for *bssA* of betaproteobacterial and deltaproteobacterial degraders were applied to different monitoring wells of a former coal gasification plant in Glassboro, New Jersey, showing that degrader abundance was enriched by several orders of magnitude in central zones of the plume (Oka *et al.*^2011^).

Genes for the central enzymes of anaerobic monoaromatic compound degradation have also been employed to monitor degraders in the field. Hence, a substantial diversity of class I benzoyl-CoA reductase (*bcrA*) genes was first reported for a gasoline-contaminated aquifer in Kumamoto, Japan (Hosoda *et al.*^2005^). Distinct gene pools were found in different wells and degrader abundance appeared linked to the level of contamination. Similarly, Fahrenfeld *et al.* (2014) have reported a quantitative enrichment of class I BCR (*bzdN*) genes of facultative anaerobes close to the crude oil spill at the Bemidji aquifer, Minnesota. At the Banisveld site, genes for the aromatic ring-cleaving hydrolase (*bamA*) were monitored parallel to *bssA* (Staats, Braster and Röling 2011). While the gene pool of *bamA* was considerably more diverse than that of *bssA*, it was surprisingly more abundant outside the plume. A comprehensive qualitative assessment of catabolic gene pools for monoaromatics degradation (*bcrC*, *bamB* and *bamA*) has been performed for samples from two aquifers with high (Ruhr area) or low (Gneisenau) benzene contamination in Germany (Kuntze *et al.*^2011^). The differentiation between mostly beta- and deltaproteobacterial degraders was largely consistent for both sites.

Apart from these central enzymes, studies tracing peripheral markers of benzene degradation, such as anaerobic benzene carboxylase (AbcDA, Abu Laban *et al.*^2010^; Holmes *et al.*^2011^), have not been published to date. While such assays should certainly be developed, the detection of central catabolic markers remains an essential tool to characterise benzene degraders in anoxic aquifers. It must be noted, however, that benzoyl-CoA is a central intermediate not only in the anaerobic degradation of BTEX hydrocarbons, but also in the degradation of humics, aromatic amino acids and lignins (Porter and Young 2014). Therefore, the detection of such central markers may not always be strictly linked to populations actually involved in anaerobic aromatic hydrocarbon degradation.

Obviously, all of these gene assays have also been applied to aromatics-degrading laboratory microcosms and enrichments. The detectability of betaproteobacterial *bssA* was first reported for toluene-degrading aquifer enrichments under denitrification (Beller *et al.*^2002^). An unidentified betaproteobacterial *bssA* lineage was first reported for iron-reducing, toluene-degrading enrichments from the Banisveld aquifer (Botton and Parsons 2007) and later identified to represent the novel isolate *G. toluolica* (Weelink *et al.*^2009^). *BssA* genes of desulfobulbal affiliation were detected in both toluene- and xylene-degrading sulfidogenic enrichments from the BTEX-contaminated Zeitz aquifer, Germany (Herrmann *et al.*^2009^; Jehmlich *et al.*^2010^). Deeply branching *bssA* homologues related to the ‘T-cluster’ FAE genes first detected in the field (Fig. 3) were also reported for the xylene microcosms, again demonstrating vital feedbacks between research performed in lab and field.

The expression of a novel and deeply branching *bssA* phylotype was first demonstrated by Washer and Edwards (2007) for a methanogenic, toluene-degrading enrichment from the Pensacola aquifer, Florida. In this culture, *Desulfotomaculum* spp. had been identified as an important component, giving a first hint at the phylogenetic placement of clostridial *bssA* (Fig. 3). More recently, related *bssA* lineages of members of the *Peptococcaceae* have also been reported for a methanogenic, toluene-degrading enrichment from the Fort Lupton aquifer (Fowler *et al.*^2012^, ^2014^) and a number of peptococcal genomes (Poehlein, Daniel and Simeonova 2013; Kuever *et al.*^2014^; Abu Laban *et al.*^2015^). This substantiates the affiliation of these deeply branching *bssA* sequence types. Evidence for niche partitioning between distinct *bssA* gene pools has been elaborated for sulfidogenic toluene-degrading enrichments from the Zeitz aquifer (Kuppardt *et al.*^2014^). Here, desulfobulbal *bssA* dominated enrichments from less contaminated zones, while peptococcal *bssA* was found in those from highly contaminated inocula. For the comprehensive detection of such peptococcal *bssA* genes and other more deeply branching FAE genes, optimised primer systems have recently been developed (von Netzer *et al.*^2013^).

Amongst the different central catabolic markers, aromatic ring-cleaving *bamA* hydrolase genes have been detected in a number of sulphate-reducing enrichments from the Zeitz aquifer (Kuntze *et al.*^2008^). More recently, the expression of benzene carboxylase transcripts and no less than three distinct pathways for benzoyl-CoA catabolism were proven for nitrate-reducing enrichments from a gasoline-contaminated aquifer in Toronto, Canada (Luo *et al.*^2014^). Taken together, the studies summarised here demonstrate how catabolic marker gene detection in lab and field can provide valuable cues for the interpretation of degrader identity and diversity in contaminated systems.

### Isotopic labelling of degraders

A second major strategy to trace degraders of BTEX hydrocarbons in contaminated aquifers relies on the isotopic labelling of biomarkers via labelled substrates. This so-called SIP approach can be performed for different cellular biomarkers such as phospholipid fatty acids, nucleic acids, proteins and even entire cells and is widely applied in lab and field. A detailed comparison of the strengths and limitations of the different methodologies and their application to anaerobic hydrocarbon degraders is beyond the scope of this review, but available elsewhere (Vogt *et al.*^2016^). Nucleic acid- and protein-based SIP especially have been utilised to characterise anaerobic degraders of BTEX pollutants in aquifer materials, or in related laboratory enrichments. While the results of the first may be more directly linked to processes in the field, the probing of cultures can provide more detailed insights into interactions and carbon sharing within degrader communities (Kleinsteuber, Schleinitz and Vogt 2012; Vogt *et al.*^2016^).

For groundwater samples, rRNA-SIP was first applied to identify denitrifying benzene degraders at a gasoline-contaminated aquifer in Kumamoto, Japan (Kasai *et al.*^2006^). The identified betaproteobacterial degraders belonged to the genus *Azoarcus* and could be subsequently isolated in pure culture. Anaerobic benzene degraders were also traced by DNA-SIP at the coal tar-contaminated South Glens Falls aquifer, New York (Liou, DeRito and Madsen 2008). Sediment microcosms incubated under comparative electron acceptor amendments and direct labelling in the field revealed a remarkable diversity of labelled taxa. Most importantly, the betaproteobacterial *Pelomonas* spp. was consistently labelled. In a follow-up study combining DNA-SIP and metagenomics, denitrifying toluene degraders from the same site were identified as *Herminiimonas* spp. (*Betaproteobacteria*), providing a wealth of detail on the catabolic potentials and adaptations of these degraders to the site (Kim *et al.*^2014^).

Sulphate-reducing toluene degraders related to *Desulfosporosinus* spp. were first identified by DNA-SIP in samples from the tar oil-contaminated Testfeld Süd aquifer (Winderl *et al.*^2010^). Via *bssA* fragments retrieved from ^13^C-labelled DNA, the authors provided labelling-assisted evidence for the peptococcal affiliation of the previously unidentified F2-cluster *bssA* (Fig. 3). Similarly, DNA-SIP suggested the F1-cluster *bssA* genes first detected at the Flingern site to be linked to degraders within the *Desulfobulbaceae* (Pilloni *et al.*^2011^). This was surprising, given the closer affiliation of this lineage with geobacterial *bssA* than that of other *Desulfobulbaceae* (Fig. 3) and suggests a possible role of horizontal transfer in local community adaptation to pollution.

Other researchers have used SIP to identify degraders in laboratory cultures previously enriched from contaminated aquifers, often maintained in the lab for a considerable time span. In sulphate-reducing enrichments form the Zeitz aquifer, DNA-SIP identified members of the *Desulfobulbaceae* as key toluene degraders (Bombach *et al.*^2010^), and distinct *Peptococcaceae* (*Pelotomaculum* and *Cryptoanaerobacter* spp.) as primary oxidisers of benzene (Herrmann *et al.*^2010^). In a pioneering follow-up study involving protein-SIP, the role of *Peptococcaceae* was also confirmed via labelled peptides (Taubert *et al.*^2012^). This approach led to a conceptual model of organic and inorganic carbon flow between primary degraders and secondary community members, as will be discussed further down. Protein-SIP has also been used to probe the degradation of m-xylene in a sulphate-reducing enrichment from the Zeitz aquifer (Bozinovski *et al.*^2012^). Peptides of proteins involved in sulphate reduction, xylene oxidation and C1 metabolism in members of the *Desulfobacteraceae* were identified as ^13^C labelled. Finally, the aforementioned methanogenic, toluene-degrading enrichment culture from the Fort Lupton aquifer has recently been dissected using rRNA-SIP (Fowler *et al.*^2014^). Here, *Desulfosporosinus* spp. was revealed as most important assimilator of ^13^C label.

One general finding of the above SIP studies was the low efficiency of assimilation of ^13^C from fully labelled BTEX compounds by strictly anaerobic degraders. Typically, this was only ∼50% (Bombach *et al.*^2010^; Herrmann *et al.*^2010^; Winderl *et al.*^2010^; Pilloni *et al.*^2011^; Taubert *et al.*^2012^), pointing at substantial ratios of assimilated carbon not stemming from the contaminant. This was also directly proven by comparative SIP labelling of the above sulphate-reducing, benzene-degrading enrichment with ^13^CO_2_ (Rakoczy *et al.*^2011^). This substantial heterotrophic CO_2_ fixation has been attributed to the fact that strictly anaerobic degraders within the *Deltaproteobacteria* or *Peptococcaceae* typically lack the glyoxylate cycle and thus assimilate acetyl-CoA through reductive carboxylation (Winderl *et al.*^2010^). Alternatively, evidence for parallel ongoing CO_2_ fixation by key degraders via the Wood-Ljungdahl pathway has also been provided (Taubert *et al.*^2012^). These exceptionally high ratios of inorganic carbon assimilation during heterotrophic contaminant degradation need to be taken into account in the interpretation of labelling results for strictly anaerobic degraders, especially if secondary carbon sharing also occurs.

In summary, this SIP work suggests not only a primary relevance of distinct *Beta*- and *Deltaproteobacteria* in BTEX-contaminated aquifers, but also of the *Peptococcaceae*. This is consistent with labelling results obtained for samples from other terrestrial systems, such as contaminated soils and sediments (Kunapuli, Lueders and Meckenstock 2007; Sun and Cupples 2012; van der Zaan *et al.*^2012^; Sun, Sun and Cupples 2014) or oil sands (Abu Laban, Dao and Foght 2015). Besides primary degraders, these studies have also revealed a complex and often overlapping accessory microbiome, including other *Deltaproteobacteria*, *Epsilonproteobacteria*, *Bacteroidetes* and also *Archaea.* Recent insights into the patterns of carbon and electron sharing in this ‘team play’ of anaerobic aromatics degradation (Kleinsteuber, Schleinitz and Vogt 2012) will be discussed in the following.

## ECOLOGICAL CONTROLS OF DEGRADER POPULATIONS AND THEIR ACTIVITY *IN SITU*

### Substrate concentrations

As summarised in the previous section, diverse groups of anaerobic degraders of BTEX aromatics have been identified as relevant in contaminated aquifers. A discussion of their ecology as well as the controls of their activities *in situ* thus becomes possible, including interactions and feedback mechanisms between degraders and their abiotic as well as biotic environment. Bioavailability is not considered a central limiting factor in the degradation of BTEX compounds due to their good water solubility. In contrast, lower threshold concentrations for anaerobic degradation of monoaromatic hydrocarbons have only rarely been addressed, both in pure culture and in degrader communities. Toluene can be ubiquitous in non-contaminated aquatic systems (in nM background concentrations) due to its formation during the anaerobic degradation of aromatic amino acids (Fischer-Romero, Tindall and Jütner 1996). Threshold concentrations for degradation in sediment microcosms have been reported in the range of ∼1 μM for benzene and ethylbenzene, while they were at least two orders of magnitude lower for toluene and xylenes (Cozzarelli *et al.*^2010^). Still, it is unknown whether high-affinity degraders actually exist. Rather, BTEX utilisation at low concentration is believed to occur in cometabolism, e.g. under the simultaneous utilisation of all substrates potentially available to a given degrader. For two anaerobic toluene degraders, it has been shown that a variety of uptake systems or enzymes involved in the catabolism of diverse aromatic and aliphatic substrates (not actually present in the cultures) were upregulated during slow growth under substrate limitation (Trautwein *et al.*^2012^; Marozava *et al.*^2014^). This can be seen as an ‘alertness’ of the degraders to consume additional substrates, should they transiently become available.

At high BTEX concentrations, toxic effects on aquifer microbes including degraders are possible. As for other hydrocarbons, BTEX compounds tend to accumulate in cellular membranes, increasing membrane fluidity (Heipieper and Martínez 2010). For a number of typical anaerobic degraders, effective concentrations resulting in 50% growth inhibition (EC50) have been reported in the range of ∼0.2 mM for ethylbenzene and xylene, ∼0.5 mM for toluene and 1.5 mM for benzene (Duldhardt *et al.*^2007^). Although the solubility of BTEX compounds in complex mixtures often found at contaminated sites is lowered, maximal pollutant concentrations above this range have been reported (Winderl *et al.*^2008^; Tischer *et al.*^2013^; Kuppardt *et al.*^2014^). Thus, toxic effects on *in situ* degrader populations have to be taken into account, but require further elucidation.

### Electron acceptors

The availability of electron acceptors is widely recognised as a critical control for anaerobic degrader activity (Meckenstock *et al.*^2015^). The high organic carbon loads in hydrocarbon plumes can be sufficient to deplete all electron acceptors available in the water or sediment matrix. Although methanogenic degradation of monoaromatics is thermodynamically feasible (Table 1) and has been reported for a number of laboratory enrichments (Edwards and Grbić-Galić 1994; Ficker *et al.*^1999^; Ulrich and Edwards 2003; Fowler *et al.*^2012^), methanogenic degrader ecology has rarely been studied in the field (Tischer *et al.*^2013^; Fahrenfeld *et al.*^2014^). The comparative contribution of methanogenic degradation to overall biodegradation processes has been questioned (Bekins *et al.*^2001^), but methanogenic contaminated aquifers are widespread and certainly await a more fundamental investigation. Especially, possible constraints of groundwater flow on the obligate syntrophic interactions involved remain to be elaborated.

Even if electron acceptors are mostly depleted in the plume core, they are continuously replenished with groundwater flow by advective and dispersive mixing at the plume fringes. This very limited mixing of electron donors and acceptors in porous media has been recognised as one of the key constraints of anaerobic hydrocarbon degradation *in situ* (Meckenstock *et al.*^2015^). If sampled at appropriate resolution, contaminant plumes are indeed characterised by steep vertical counter gradients of electron acceptors and donors at the fringes (van Breukelen and Griffioen 2004; Tuxen, Albrechtsen and Bjerg 2006; Anneser *et al.*^2008^; Tischer *et al.*^2013^), giving rise to characteristic hot spots, or rather hot zones of biodegradation *in situ*. Especially at the Flingern site, substantial work has been done to characterise these hot spots, revealing multiple lines of evidence for enhanced biodegradation activity and a quantitative enrichment of specific degrader lineages, respective catabolic genes and metabolites at the plume fringes (Winderl *et al.*^2008^; Jobelius *et al.*^2010^; Larentis, Hoermann and Lueders 2013; Einsiedl *et al.*^2015^). Degrader enrichment also correlated with a decreased overall community diversity in hot spots (Winderl *et al.*^2008^), which was in line with the perspective that a high functional organisation of microbial communities can support a most efficient catalysis of specific biogeochemical processes (Marzorati *et al.*^2008^).

### Degrader diversity

General ecological theory applied to microbial systems suggests that overall community diversity and evenness should be directly related to functional stability upon disturbance (Shade *et al.*^2012^). Moreover, the number of functionally redundant species specifically (or potentially) active in a given process can be a measure of system resistance and resilience against disturbance (Konopka 2009). Overall microbial diversity in BTEX plumes has been frequently characterised, and several studies report clear shifts in microbial diversity between plume zones and even increased diversity in highly contaminated samples (Feris *et al.*^2004^; Fahy *et al.*^2005^; Staats, Braster and Röling 2011; Tischer *et al.*^2013^; Fahrenfeld *et al.*^2014^). However, highly selected catabolic gene pools and thus a low functional redundancy of anaerobic toluene degraders have also been reported for several sites (Winderl, Schaefer and Lueders 2007; Callaghan *et al.*^2010^; Staats, Braster and Röling 2011). For these apparently ‘specialised’ degrader communities, a low functional stability upon habitat disturbance could be implied. Although aquifers are classically considered as steady-state habitats, the impact of hydraulic fluctuations and resulting rearrangements in contaminant distribution and biogeochemical regime are considered a key unknown in aquifer restoration (Qiu 2011). Haack *et al.* (2004) showed that recharge-connected variations in the groundwater table can be connected to profound rearrangements of overall microbial community structure and to a loss of diversity in specific zones of the BTEX-contaminated Wurtsmith aquifer, Michigan. Although not for a BTEX plume, significant changes of aquifer microbiota driven by fluctuating river water intrusions and related nutrient and electron acceptor fluxes have been reported for the uranium-contaminated Hanford aquifer, Washington (Lin *et al.*^2012^). Such couplings between hydraulic and biogeochemical habitat dynamics and the activity of degrader populations should be investigated more extensively in the future. For sites where a larger diversity of *bssA* genes was found (Yagi *et al.*^2010^), it is still unclear whether this indicates functional redundancy amongst degraders, or whether this simply reflects a more complex setting of contamination or redox scenarios at the given site. Indeed, substrate-dependent clustering of *bssA* gene pools has been reported (Acosta-González, Rosselló-Móra and Marqués 2013; Jarling *et al.*^2015^).

### Respiratory versatility

One mechanism for degrader populations to cope with redox fluctuations would be the utilisation of variable electron acceptors. It is interesting to consider that although all known denitrifying toluene degraders are facultative anaerobes, only two strains have been reported to host both aerobic and anaerobic catabolic pathways (Shinoda *et al.*^2004^; Martín-Moldes *et al.*^2015^). For a facultative anaerobic degrader population established at the fringe of a BTEX plume, it would be advantageous to utilise either aerobic or anaerobic catabolism depending on oxygen availability. However, since nature does not seem to have evolved many such degraders, other adaptive mechanisms could be in place. Potentially, degrader niches at fluctuating oxic/anoxic redox gradients could be filled by ‘aerobic’ degraders alone. The discovery of the denitrifying methanotroph ‘*Methylomirabilis oxyfera’* isolated from a Dutch canal (Ettwig *et al.*^2010^) proposes a possible mechanism. Self-sustained oxygenesis via NO dismutation allows this microbe to oxidise methane using aerobic catabolic pathways in redox compartments where molecular oxygen is depleted, but where nitrate or nitrite are available. An analogous monooxygenase-dependent catabolism of alkanes has been suggested for the denitrifying *Gammaproteobacterium* HdN1 (Zedelius *et al.*^2011^) and for the benzene-degrading denitrifying *Dechloromonas aromatic* (Weelink, van Eekert and Stams 2010). Such physiology could perhaps explain the surprisingly high abundance of toluene monooxygenase genes detected in the anoxic plume core of the BTEX-contaminated Flingern aquifer (Larentis, Hoermann and Lueders 2013).

Furthermore, the discovery of ‘cable bacteria’, i.e. marine *Desulfobulbaceae* capable of coupling sulphide oxidation to oxygen respiration via filamentous electron transfer over centimetre distances (Pfeffer *et al.*^2012^) may have implications for contaminated aquifers. Members of the *Desulfobulbaceae* have not only been identified as dominating anaerobic degrader populations at a number of BTEX-contaminated sites (Bombach *et al.*^2010^; Callaghan *et al.*^2010^; Pilloni *et al.*^2011^; Sun and Cupples 2012), they have also recently been shown capable of long-distance electron transfer in Flingern aquifer sediments (Müller *et al.*^2016^). Besides an indirect role in electron acceptor recycling via electrogenic sulphide oxidation, it remains to be shown whether cable bacteria could also have a direct role in hydrocarbon degradation at plume fringes. This adaptation would allow classical sulphate-reducing degraders to extend their habitat over oxic/anoxic redox gradients, and to maintain anaerobic BTEX catabolism while actually respiring oxygen and thus exploiting much more favourable thermodynamics (Table 1). Apart from these recent discoveries, deltaproteobacterial *Geobacter* spp. are also known for their versatile use of different anaerobic electron acceptors including metal oxides, nitrate and humics (Coates *et al.*2001a). This may support their activity under varying redox conditions *in situ*.

### Sharing of labour

Syntrophic interactions and other mechanisms of carbon and electron sharing are recognised as a key feature in anaerobic hydrocarbon degradation, not only in methanogenic systems (Weelink, van Eekert and Stams 2010; Kleinsteuber, Schleinitz and Vogt 2012; Gieg, Fowler and Berdugo-Clavijo 2014). Ulrich and Edwards (2003) reported that several benzene-degrading enrichments could readily switch between sulphate-reducing and methanogenic conditions, suggesting that identical initial degraders were active in both. A role of syntrophy has also been inferred via SIP for several iron-reducing (Kunapuli, Lueders and Meckenstock 2007), sulphate-reducing (Herrmann *et al.*^2010^; Taubert *et al.*^2012^) and even denitrifying (van der Zaan *et al.*^2012^) benzene-degrading enrichments. All were dominated by primary degraders within the *Peptococcaceae*, and their sharing of hydrogen or electrons with syntrophic partners within the *Deltaproteobacteria* or *Rhodocyclaceae* is suggested. The latter culture could also rapidly switch to the reduction of iron or sulphate, suggesting that the same initial benzene degraders could interact with different partners (van der Zaan *et al.*^2012^).

It is not yet fully understood whether the primary degraders in such consortia essentially act as fermenters, as incomplete substrate oxidisers or even a mixture of both. The former would be supported by the lack of dissimilatory sulphate reduction genes recently reported for a *Desulfosporosinus* sp. from a toluene-degrading methanogenic enrichment culture (Abu Laban, Dao and Foght 2015; Abu Laban *et al.*^2015^). As shown in Table 1, the incomplete oxidation of toluene to acetate by a sulphate-reducing degrader would also be thermodynamically feasible, especially if calculated per mol of sulphate, much more than the fermentation to acetate and hydrogen. Potentially, incomplete oxidation could be more sustainable in habitats with limited electron acceptor supply such as contaminant plume fringes. However, true syntrophy with sharing of hydrogen (or electrons), acetate and other fermentation products is also supported for some of the above enrichment cultures. Inhibitory effects of both hydrogen and acetate amendment on anaerobic benzene degradation have been shown (Rakoczy *et al.*^2011^; van der Zaan *et al.*^2012^) and respective protein-SIP work provides evidence for a role of acetate as shared metabolite (Taubert *et al.*^2012^; Starke *et al.*^2016^). A role of syntrophy in toluene oxidation has also been suggested for systems dominated by *Geobacteraceae* (Botton *et al.*^2007^). Here, Meckenstock (1999) reported that *Geobacter* can degrade toluene in syntrophy with a fumarate-reducing *Wollinella*, despite *Geobacter's* inability to reduce fumarate alone. Potentially, direct interspecies electron transfer (Wang *et al.*^2016^) could also play a role in syntrophic BTEX degradation by *Geobacteraceae*.

Apart from direct syntrophies, indirect interactions and patterns of carbon sharing also seem to be important in the anaerobic degradation of BTEX compounds. Distinct *Epsilonproteobacteria* related to *Sulfurovum* and *Sulfuricurvum* spp. are notoriously detected in respective enrichments and at contaminated sites (Kleinsteuber *et al.*^2008^; Herrmann *et al.*^2010^; Pilloni *et al.*^2011^; Bozinovski *et al.*^2012^; Einsiedl *et al.*^2015^). Such *Epsilonproteobacteria* are typically known as chemolithoautotrophic sulphide oxidisers respiring oxygen or nitrate. While their physiology in anaerobic hydrocarbon-degrading consortia is not yet fully understood, recent evidence suggests potentials roles as acetate scavengers and in polysulfide cycling (Keller *et al.*^2015^; Starke *et al.*^2016^). Taken together, syntrophic interactions and other mechanisms of carbon and electron sharing seem to be an almost universal feature of microbial consortia active in anaerobic aromatics degradation.

## BIOREMEDIATION AND AQUIFER RESTORATION

As reviewed above, considerable advances of our understanding of the diversity and ecology of anaerobic degraders of BTEX hydrocarbons in aquifers have been accomplished. It remains to be discussed how this can actually benefit the management and restoration of contaminated sites. Such benefit should be apparent on two levels: that of monitored natural attenuation and that of bioremediation. The monitoring of natural attenuation involving tools of molecular environmental microbiology is still far from routine. Wilson, Thornton and Mackay (2004) suggested that monitoring should focus on the localisation of key degradation activities in critical zones of a contaminated aquifer. Spatially resolved sampling is thus a crucial issue which may not always be readily accomplished. In today's routine monitoring schemes, depth-resolved sampling of aquifers is done at metre rather than at centimetre scales, if at all. Spatial heterogeneity is thus not assessed at relevant resolution, and hot spots of biodegradation may be missed (Meckenstock *et al.*^2015^).

Still it is clear that catabolic gene detection offers an unparalleled means to localise and quantify degraders at contaminated sites. The à priori identification of dominating degrader populations, e.g. by SIP, can be an important prerequisite (Vogt *et al.*^2016^). A considerable number of field studies have included quantitative marker gene-based monitoring of the spatial distribution or temporal development of anaerobic degraders (Hosoda *et al.*^2005^; Beller *et al.*^2008^; Winderl *et al.*^2008^; Oka *et al.*^2011^; Staats, Braster and Röling 2011; Fahrenfeld *et al.*^2014^). A direct correlation between *bssA* gene abundance and anaerobic toluene degradation rates has been reported for aquifer microcosms (Kazy, Monier and Alvarez 2010). However, it must be remembered that quantitative gene detection allows assessing established potentials, and not actually ongoing degradation activities. Therefore, the detection and quantification of respective catabolic gene transcripts is of special interest for the assessment and prediction of biodegradation rates (von Netzer *et al.*^2016^). The quantitative monitoring of *bssA* transcript-to-gene ratios has indeed been shown to reflect anaerobic toluene degradation activities in an indoor model aquifer (Brow *et al.*^2013^). However, the incorporation of more advanced metagenomic, transcriptomic or even metabolomic approaches are still far from routine in contaminated site monitoring (Callaghan 2013; Tan *et al.*^2015^).

The identification, localisation and quantification of intrinsic degraders can also guide the development of site-specific restoration strategies. Molecular monitoring results can assist targeted amendments of electron acceptors or nutrients, or help to evaluate degrader amendments in bioaugmentation (Kasai *et al.*^2007^). Any enhanced site management scheme based on direct knowledge on intrinsic degraders will be an important step towards more integrated concepts in bioremediation. Optimally, such schemes will be based on multiple lines of evidence for active degradation processes, including biogeochemical monitoring, contaminant stable isotopes, molecular markers and metabolites (Callaghan 2013; Meckenstock *et al.*^2015^; von Netzer *et al.*^2016^).

## CONCLUSIONS AND PERSPECTIVES

This review summarises recent advances into the diversity and ecology of anaerobic degraders of BTEX hydrocarbons in contaminated aquifers. A number of novel or previously unrecognised degrader lineages within the *Desulfobulbaceae*, the *Rhodocyclaceae* and the Gram-positive *Peptococcaceae*, which are still only poorly represented within our culture collections, have been identified to dominate the degradation of monoaromatic pollutants at a range of different sites. However, it has become clear that in most settings, complex microbial interactions or even obligate syntrophic sharing of labour will be involved in anaerobic hydrocarbon degradation *in situ*. Thus, not only a key-player perspective, but also a microbiome perspective becomes very relevant to understand pollutant degradation processes in complex natural systems. A more systematic application of latest ‘meta-omics’ approaches directly at contaminated sites will facilitate further insights into new ecophysiologies and the microbial interaction networks involved, and how they can potentially be employed in a population-based site restoration.

The perspective of the porous medium as microbial habitat, with all its special features and limitations, is now also recognised as a key control of degrader populations and their activity. Hydraulic and geochemical regimes define windows of opportunity in which degradation can take place. However, hot spots and also hot moments of biodegradation activity are currently still only rarely considered in site monitoring and reactive transport modelling schemes. Even though BTEX compounds are typically considered as legacy contaminants and many sites have been contaminated for decades or centuries, the detailed understanding of the microbes and processes controlling their degradation which is now available make them ideal model systems to test how general ecological theories such as disturbance ecology or redundancy concepts apply to contaminated systems. This will not only help to develop stronger predictions of possible feedbacks of climate change and changing land use on groundwater quality, it will also be valuable to safeguard groundwater resources against contaminants of emerging concern. Thus, the anaerobic degradation of BTEX hydrocarbons will continue to serve as a prime model system to generate a generic ecological and mechanistic understanding of contaminant degradation processes in groundwater.
